# Direct quantification of waterborne viruses via high-temperature and high-pressure treatment: a simplified nucleic acid extraction-free approach

**DOI:** 10.1038/s41598-026-57432-2

**Published:** 2026-06-21

**Authors:** Keita Soda, Yuki Miyauchi, Hiroyuki Katayama, Yasuhiro Matsui

**Affiliations:** 1 Yokogawa Electric Corporation, GX Business Development Department, Water Reuse Business Development Section, Tokyo, Japan; 2 The University of Tokyo, Graduate School of Engineering, Department of Urban Engineering, Tokyo, Japan

**Keywords:** Biological techniques, Biotechnology, Environmental sciences, Microbiology, Molecular biology

## Abstract

High-temperature and high-pressure treatment (HTP) is a physical lysis method that enables direct quantification of viral genomes from environmental samples without nucleic acid extraction. This study assessed the applicability of HTP for rapid virus detection using pepper mild mottle virus (PMMoV) as a model. Optimal conditions for PMMoV quantification were identified as 120 °C for 15 s, yielding results comparable to conventional RNA extraction methods. RNA integrity analysis revealed that temperatures above 140 °C may cause genome degradation, supporting the selection of 120 °C as the optimal setting. The method demonstrated high reproducibility across qPCR and dPCR assays and enabled stable quantification with minimal interference from wastewater matrix components. In addition, additional virus species were evaluated to assess the performance of HTP beyond PMMoV under the tested conditions. These findings highlight the potential of HTP as a simplified workflow without nucleic acid extraction for environmental virus monitoring. Future research should focus on expanding its use to diverse virus types and integrating the method into automated platforms for real-time monitoring.

**Supplementary Information:**

The online version contains supplementary material available at 10.1038/s41598-026-57432-2.

## Introduction

Pepper mild mottle virus (PMMoV) has emerged as a promising surrogate indicator for enteric viruses in environmental monitoring, particularly in the context of water reuse and wastewater-based epidemiology^1^. Its high environmental stability and frequent detection in human-impacted water sources make PMMoV a reliable marker for fecal contamination^1–3^.

Standard viral quantification workflows typically involve nucleic acid extraction and purification prior to RT-qPCR (ISO 15216-1:2017). In environmental samples, this process can be time-consuming and susceptible to inhibition of enzymatic reactions such as PCR, due to the presence of inhibitory substances in complex sample matrices^4–6^. To address these limitations, simplified approaches that bypass nucleic acid purification such as direct RT-qPCR following viral lysis have gained attention for their speed and practical applicability^7–9^.

In this study, we applied high-temperature and high-pressure treatment (HTP) to disrupt viral particles and enable direct quantification without nucleic acid purification. HTP is a physical lysis method originally developed for lysing cells, bacteria, and fungi (Ozeki et al. US Patent 8877918 B2 (2014)). The HTP device (Supplemental Fig. S1), developed in-house and not commercially available, utilizes a specialized pressure-resistant tube to expose samples to approximately 140 °C and 0.4 MPa. The key parameters for effective lysis are rapid heating, short exposure time, and pressure retention during the process. Preliminary in-house evaluations suggest that this high-temperature and high-pressure environment effectively lyses pathogenic microorganisms. This may enable reliable nucleic acid quantification from small sample volumes. We hypothesized that HTP could also effectively lyse viral particles, exposing their genomes for direct RT-qPCR analysis.

Given that qPCR targets only short regions of the genome, it may not fully reflect the overall integrity of RNA^10^. This is particularly relevant in environmental samples, where RNA degradation is common^11–13^. Therefore, we also considered the potential impact of HTP on RNA fragmentation and its implications for quantification accuracy.

Here, we demonstrate that HTP enables rapid and reliable quantification of viral genomes directly from wastewater samples without nucleic acid extraction. To validate the quantification performance of the HTP, both RT-qPCR and digital PCR (dPCR)^14^ were used in a complementary manner, allowing cross-verification of results across a wide concentration range. This method offers a promising approach for simplified environmental virus monitoring workflows.

## Results

### Effects of temperature and heating duration on PMMoV quantification

Viruses in wastewater were first concentrated to prepare virus-concentrated samples. These samples were then subjected to HTP, and the effects of heating temperature and duration on viral RNA detection were evaluated using one-step RT-qPCR, in which reverse transcription and qPCR are performed in a single tube for quantification of PMMoV.

The standard curve for one-step RT-qPCR was generated using synthetic oligonucleotides of PMMoV with known concentrations. The assay demonstrated high linearity across a wide dynamic range from 10^6^ to 1 gene copies/μL (*R*^2^ > 0.993, efficiency = 107 ± 4%). Based on the performance of the standard curve, the limit of detection (LOD) and the limit of quantification (LOQ) were estimated to be 1 copy/μL. The assay reliably detected 1 copy/μL with a 100% detection rate, and the coefficient of variation (CV) across the entire concentration range remained below 10%.

**Fig. 1:**
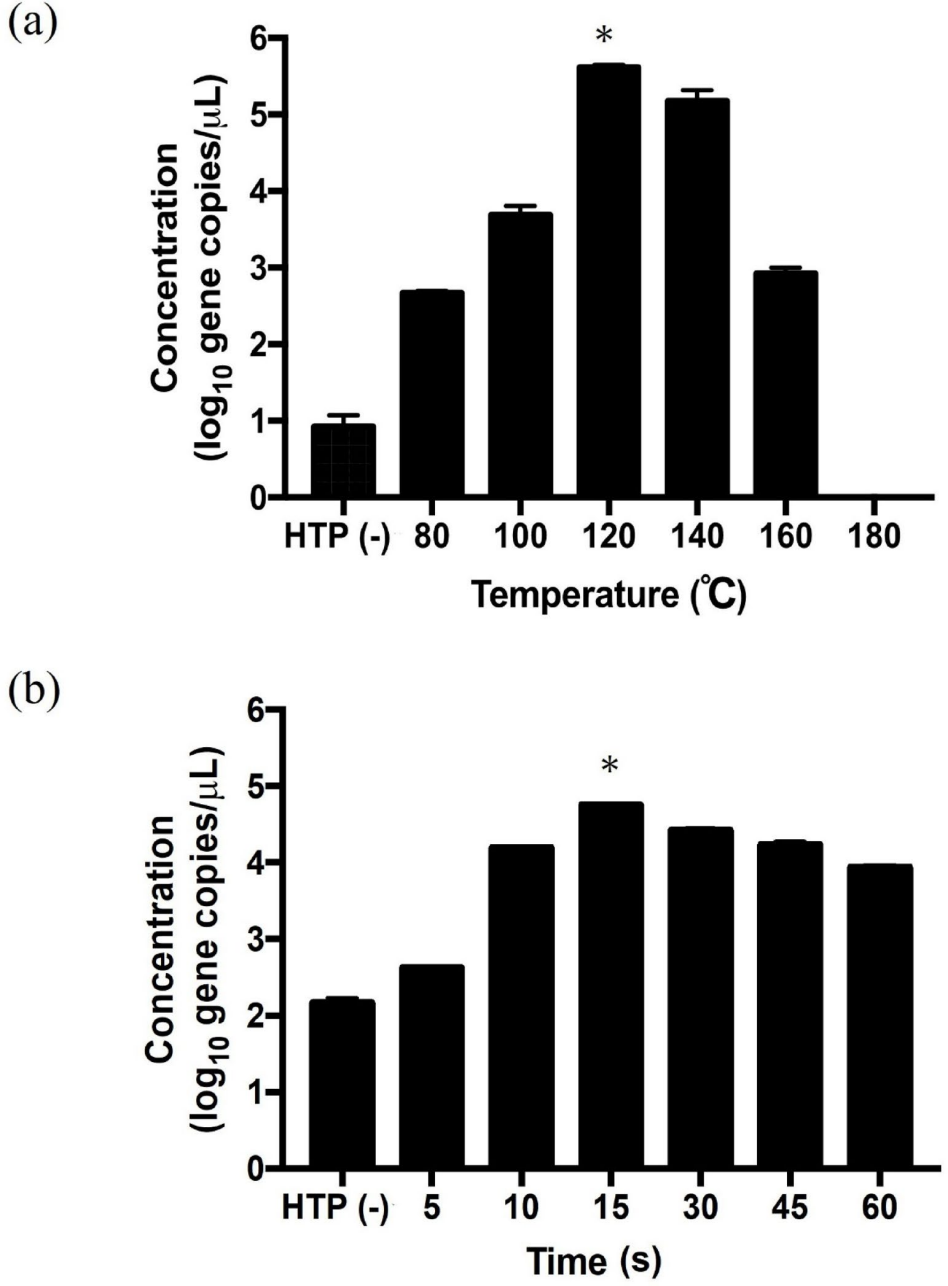
PMMoV quantification in wastewater following HTP at various temperatures and heat treatment duration. (**a**) Effect of HTP temperature on PMMoV quantification at a fixed treatment time of 15 s. (**b**) Effect of HTP treatment duration on PMMoV quantification at a fixed temperature of 120 °C. PMMoV concentrations in virus-concentrated wastewater samples were quantified under the indicated conditions. Each data point represents log_10_-transformed gene copies per μL. Error bars indicate SEM (n = 3). The highest quantification was achieved at 120 °C for 15 s (*p < 0.05, one-way ANOVA followed by Tukey’s multiple comparisons test). HTP (−) indicates virus-concentrated samples without HTP.

To assess the effect of heating temperature, HTP was performed at temperatures ranging from 80 to 180 °C in 20 °C increments. As a control, virus-concentrated samples were processed without HTP (HTP (−)), applying only the reverse transcription temperature. The results of PMMoV quantification under each condition are shown in Fig. 1. One-way ANOVA revealed that temperature had a statistically significant effect on RNA quantification (*p* < 0.05). A clear temperature-dependent trend was observed, with the highest quantification value obtained at 120 °C (5.61 log_10_ gene copies/μL, approximately 4.08 × 10^5^ gene copies/μL). At higher temperatures, quantification values sharply declined, reaching 2.92 log_10_ (837 gene copies/μL) at 160 °C and − 0.52 log_10_ (0.3 gene copies/μL) at 180 °C (Fig. 1).

To further investigate the effect of heating duration, HTP for virus-concentrated samples was performed at a fixed temperature of 120 °C, with treatment times ranging from 0 to 60 s (Fig. 1). The 0 s condition served as a control and represented the condition without HTP (HTP (−)). Thus, the observed effect reflects only the temperature applied during the reverse transcription step of the one-step RT-qPCR. PMMoV quantification increased with treatment times up to 15 s. One-way ANOVA revealed that heating duration had a statistically significant effect on RNA quantification (*p* < 0.05). Multiple comparison analysis showed that the 15-s treatment group had significantly higher values than both the 10- and 30-s groups (*p* < 0.05 for both). The mean log_10_ values were 4.20 (10 s), 4.76 (15 s), and 4.43 (30 s), with the 15-s group showing the highest value. Notably, the 10-s treatment resulted in a substantial increase—approximately 37-fold—compared to the 5-s treatment.

These results indicate that HTP treatment at 120 °C for 15 s is the optimal condition for PMMoV quantification.

### Effect of HTP on the sensitivity of PMMoV detection

To evaluate the sensitivity of the HTP method under optimized conditions (120 °C for 15 s), purified PMMoV was serially diluted from 1 × 10^4^ gene copies/μL to 1 gene copy/μL to prepare standard solutions. These samples were analyzed using both RT-qPCR and dPCR. Table 1 summarizes the mean quantification values and standard errors across six concentration levels, including a negative control. Low-level signals were occasionally detected in negative control samples; however, these signals were substantially lower than those observed in positive samples and did not affect the overall interpretation of the results. PMMoV was detectable down to 1 copy/μL by both RT-qPCR and dPCR. Reliable quantification was achieved at concentrations of 10 copies/μL and above (Supplementary Table S1). Although the coefficient of variation tended to increase at lower concentrations, detection was reproducible across all tested levels.

**Table 1:** Summary of PMMoV quantification across serial dilutions using RT-qPCR and dPCR.

Spiked PMMoV concentration (serial dilution, gene copies/μL)	RT-qPCR (gene copies/μL)	dPCR (gene copies/μL)
0 (Negative Control)	1.6 ± 0.8 (n = 6)	0.3 ± 0.1 (n = 6)
1	3.2 ± 1.0 (n = 6)	0.9 ± 0.5 (n = 6)
10	5.4 ± 0.6 (n = 3)	10.3 ± 0.9 (n = 3)
100	59.3 ± 8.4 (n = 3)	112.8 ± 1.1 (n = 3)
1000	609.0 ± 20.9 (n = 3)	916.0 ± 18.7 (n = 3)
10,000	5104.2 ± 227.3 (n = 3)	6301.1 ± 305.1 (n = 3)

Spiked PMMoV concentration refers to samples prepared by adding purified PMMoV to solution at known concentrations. Values are presented as mean ± SEM. Negative control: RNase-free/DNase-free water.

### Comparison of PMMoV genome copies between conventional RNA extraction and HTP

To evaluate the effectiveness of HTP, which eliminates the need for nucleic acid extraction, we compared it with a conventional RNA purification method using the AllPrep RNA extraction kit (QIAGEN). PMMoV genome copies were quantified under both conditions. A standardized PMMoV solution was prepared by spiking purified PMMoV into 25 mM Tris–HCl buffer.

Using PMMoV standardized viral solution, RNA extraction was performed using either the AllPrep kit or HTP. The resulting RNA or lysate was then used as the template for RT-qPCR. Both one-step RT-qPCR, where reverse transcription and amplification occur in a single tube, and two-step RT-qPCR, where cDNA synthesis is followed by qPCR, were employed for comparison.

Viral genome quantification was conducted using RNA purified with the AllPrep kit as the control under three conditions: without HTP (denoted as HTP (−), reverse-transcription–temperature only), with HTP at 80 °C, and with HTP at 120 °C, all with an HTP duration of 15 s.

**Fig. 2:**
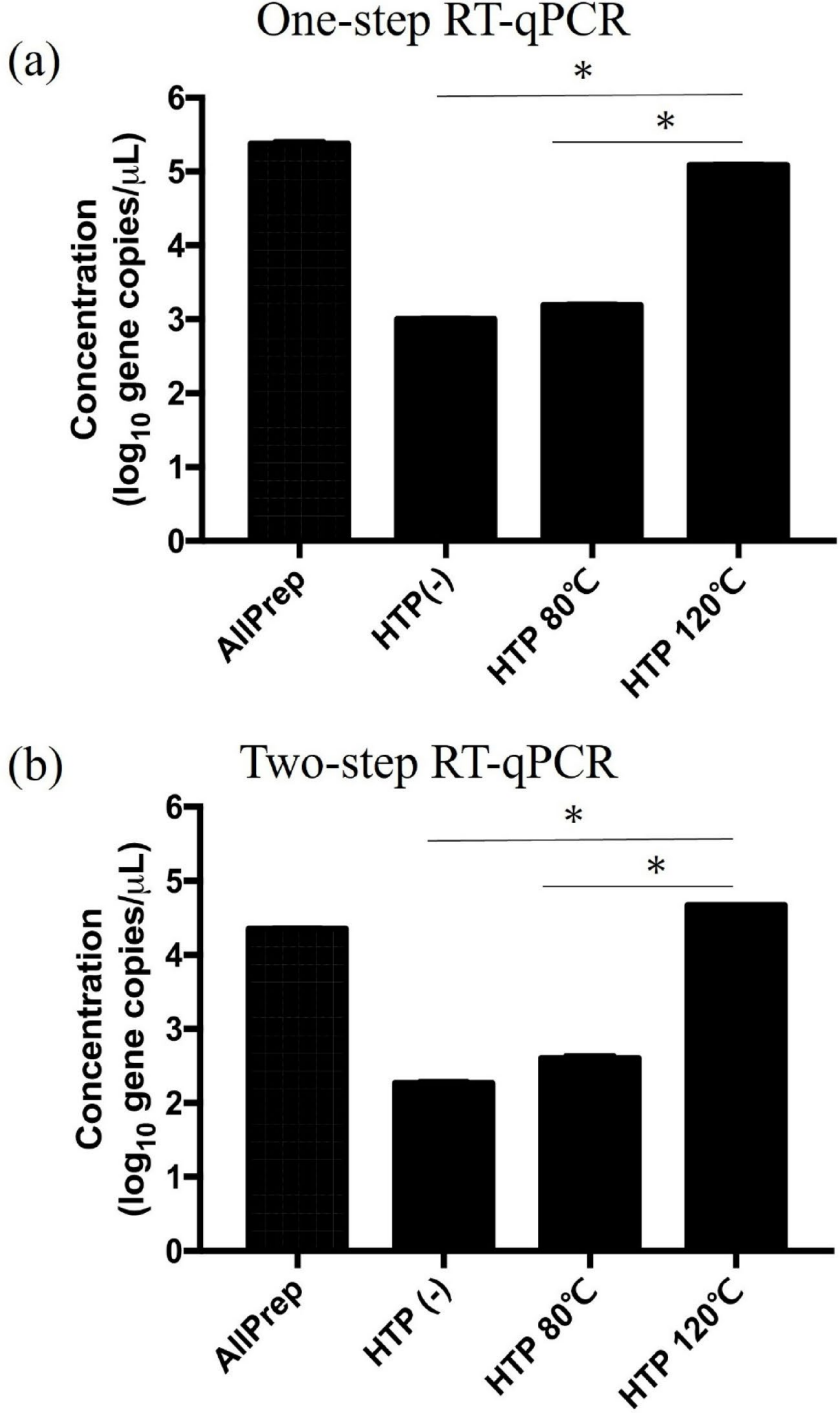
Comparison of PMMoV genome copy quantification across AllPrep and HTP conditions. The upper panel (**a**) shows results from one-step RT-qPCR, while the lower panel (**b**) presents two-step RT-qPCR data. HTP at 120 °C for 15 s yielded quantification values comparable to those obtained with RNA purified using the AllPrep kit. HTP 120 °C showed significantly higher quantification values compared to without HTP (HTP (−)) and HTP 80 °C (*p < 0.05, one-way ANOVA followed by Tukey’s multiple comparison test). Error bars indicate SEM (n = 3).

In the one-step RT-qPCR results, no statistically significant difference was observed between the quantification values obtained using the AllPrep kit and those obtained after HTP at 120 °C (*p* > 0.05), and the difference was less than one order of magnitude, indicating that the quantification scale was largely unchanged (Fig. 2). The quantification values were 5.38 log_10_ gene copies/μL for AllPrep and 5.09 log_10_ gene copies/μL for HTP 120 °C. HTP 120 °C showed significantly higher quantification values compared to HTP (−) and HTP 80 °C (3.00 and 3.19 log_10_ gene copies/μL, respectively) (*p* < 0.05), confirming the reproducibility of the trend shown in Fig. 2.

Similarly, in two-step RT-qPCR, the difference between AllPrep and HTP 120 °C was less than one order of magnitude, with quantification values of 4.35 and 4.67 log_10_ gene copies/μL, respectively (Fig. 2). HTP 120 °C yielded significantly higher values than HTP (−) and HTP 80 °C (2.27 and 2.61 log_10_ gene copies/μL, respectively; *p* < 0.05).

The standard curve for two-step RT-qPCR demonstrated high linearity (*R*^2^ > 0.997, with an amplification efficiency of 102 ± 3%) across the range of 10^6^ to 1 gene copies/μL. Measurable signals were observed down to 1 copy/μL, although variability increased at the lowest concentration. At higher concentrations, coefficients of variation (CVs) were below 10%, indicating stable quantification performance. These results confirm that two-step RT-qPCR provides reliable and precise quantification under controlled conditions.

Additionally, to validate the RT-qPCR results, dPCR was performed using the same primer set. A standardized PMMoV solution (1 × 10^5^ gene copies/μL) was quantified using RNA purified with the AllPrep kit and HTP-processed samples. As shown in Table 2, although statistically significant differences were observed between qPCR and dPCR, the differences in quantification values were 0.3 log_10_ for AllPrep and 0.4 log_10_ for HTP, remaining within the same order of magnitude. The detection of PMMoV by dPCR further supports the robustness of the HTP method.

**Table 2:** Comparison of PMMoV RNA quantification by qPCR and dPCR using AllPrep and HTP sample preparation methods.

Method	qPCR (log_10_ gene copies/μL)	dPCR (log_10_ gene copies/μL)	Log_10_ difference	*p* value (Student’s *t*-test)
AllPrep	4.4 ± 0.1	4.6 ± 0.1	0.3	0.25
HTP	4.7 ± 0.1	5.0 ± 0.1	0.4	0.35

All values are presented as mean ± SEM.

### Impact of wastewater matrix components on viral RNA detection

Virus-concentrated samples were recovered from the chip using a dedicated elution buffer, which effectively removes wastewater-derived matrix components smaller than the virus particles and replaces the buffer. A similar principle applies when using nucleic acid extraction kits such as AllPrep, where viral RNA is eluted in RNase-free H_2_O, minimizing any potential impact of the buffer on RT-qPCR reactions. In contrast, HTP method simplifies the sample preparation process for viral genome but may allow wastewater-derived matrix components to be carried over into the RT-qPCR reaction system.

To evaluate this potential interference, without HTP (HTP (−)) and with HTP (120 °C, 15 s) (HTP (+)) wastewater samples without virus concentration were quantified for PMMoV using one-step RT-qPCR. Given the expected low viral concentration, dPCR was also performed as a complementary method.

The results showed no statistically significant difference in PMMoV quantification between HTP (−) and HTP (+) samples by RT-qPCR (*p* = 0.288, n = 6, Student’s *t*-test) (Table 3) when unconcentrated wastewater samples were analyzed. Similarly, no significant difference was observed in dPCR results (*p* = 0.546, n = 6, Student’s *t*-test). These findings indicate that under low-concentration conditions, it is difficult to clearly assess the impact of wastewater matrix components carried over into the RT-qPCR reaction system by HTP treatment.

**Table 3:** Comparison of PMMoV quantification in unconcentrated wastewater samples between HTP (−) and HTP (+) conditions using qPCR and dPCR.

Method	HTP (−) (log_10_ gene copies/μL)	HTP (+) (log_10_ gene copies/μL)	*p* value (Student’s *t*-test)
qPCR	1.1 ± 0.1	1.2 ± 0.2	0.29
dPCR	0.9 ± 0.2	0.9 ± 0.5	0.55

Values are presented as mean ± SEM (n = 6).

To assess the impact of wastewater matrix components more clearly, purified PMMoV was spiked into tertiary-treated wastewater to a final concentration of 1 × 10^5^ gene copies/μL, and the samples were subjected to HTP treatment (120 °C, 15 s) followed by one-step RT-qPCR. As a control, PMMoV at the same concentration was added to 25 mM Tris–HCl buffer.

No statistically significant difference was observed in RT-qPCR detection between the spiked wastewater and buffer samples (*p* = 0.293, n = 4, Student’s *t*-test). This result demonstrates that, under the tested conditions, no inhibitory effect of wastewater matrix components carried over by HTP treatment on RT-qPCR reactions was observed (Table 4).

**Table 4:** Comparison of PMMoV quantification in spiked Tris–HCl buffer and tertiary-treated wastewater samples following HTP (n = 4).

Method	25 mM Tris–HCl (log_10_ gene copies/μL)	Wastewater (log_10_ gene copies/μL)	*p* value (Student’s *t*-test)
qPCR	5.1 ± 0.1	5.0 ± 0.1	0.29

Values are presented as mean ± SEM.

### Preservation and fragmentation of PMMoV RNA across HTP temperatures

To further validate the direct quantifiability of PMMoV RNA lysed by HTP treatment, we next evaluated its integrity. This assessment aimed to provide a more detailed understanding of RNA quality following HTP treatment. To assess the impact of thermal stress during HTP processing, RNA from lysed PMMoV samples was analyzed under various temperature conditions. Samples were subjected to electrophoresis and analyzed using a Bioanalyzer, focusing on the intensity of major RNA peaks.

**Fig. 3:**
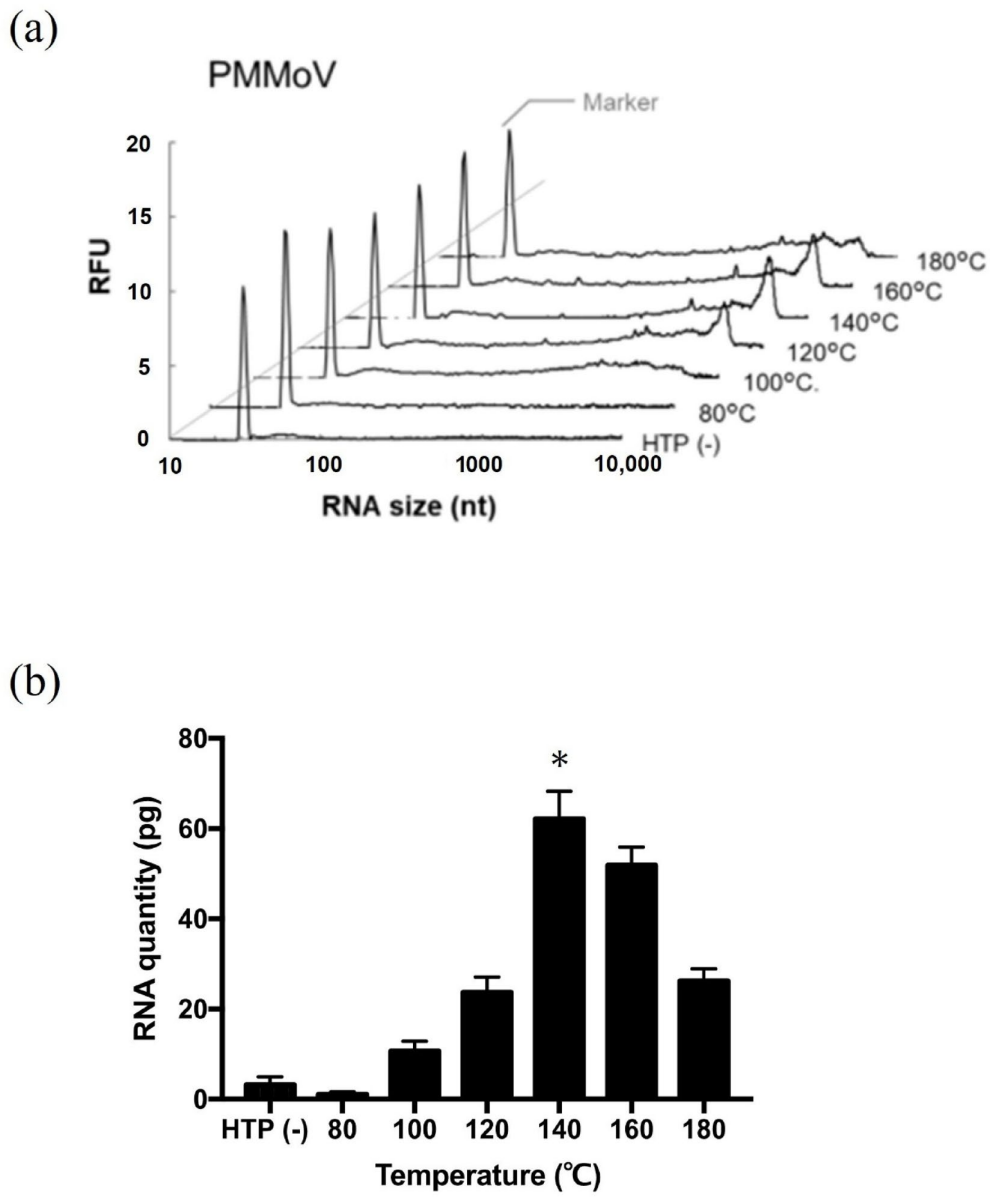
Quantification of PMMoV RNA released by HTP lysis across a range of temperatures. (**a**) Electropherogram of PMMoV RNA analyzed using a Bioanalyzer under different temperature conditions. The x-axis represents RNA size in nucleotides (nt) on a logarithmic scale, and the y-axis represents relative fluorescence units (RFU). Each trace represents RNA released under a specific HTP treatment temperature. Marker indicates 25 nucleotides (nt). (**b**) Quantification of PMMoV RNA released under different HTP treatment temperatures. RNA yield (pg) was estimated based on the intensity of electropherogram peaks within the 4000–5500 nt range, presumed to correspond to PMMoV-derived RNA. The highest RNA quantity was observed at 140 °C. HTP (−) indicates samples without HTP treatment. Error bars represent standard deviations from triplicate measurements (n = 4). RNA quantity (pg) was calculated using the Bioanalyzer based on the area under the electropherogram curve corresponding to the 4000–5500 nt range, which was presumed to represent PMMoV-derived RNA. (*p < 0.05, one-way ANOVA followed by Tukey’s multiple comparison test). Error bars indicate SEM (n = 3).

Following HTP treatment at temperatures ranging from 80 to 180 °C, distinct RNA bands were observed via electrophoresis (Fig. 3). However, these bands did not fully correspond to the full-length PMMoV genome (~ 6356 nucleotides (nt)) and were primarily located within the 4000–5500 nt range. These bands were presumed to be derived from PMMoV RNA and were used for yield evaluation. RNA quantity (picogram, pg) was estimated based on the area under the electropherogram curve corresponding to the 4000–5500 nt range, using the Bioanalyzer software.

Figure 3 shows electropherograms of RNA profiles from PMMoV lysates processed by HTP at different temperatures. The x-axis represents RNA size (nt), and the y-axis represents fluorescence intensity (RFU). Peaks within the 4000–5500 nt range were used for RNA quantification. RNA yield increased with temperature, reaching a maximum at 140 °C, and decreased at higher temperatures. One-way ANOVA revealed that differences in RNA yield across temperatures were statistically significant (*p* < 0.05). Furthermore, multiple comparison analysis indicated that the 140 °C treatment group exhibited significantly higher RNA yield compared to other temperature groups.

### Extension of HTP treatment to additional virus species

To further evaluate the applicability of HTP treatment, we examined its performance across multiple virus species with different genome types and sample types. The evaluated targets included RNA viruses such as pepper mild mottle virus (PMMoV), cucumber green mottle mosaic virus (CGMMV), norovirus genogroup II (GII), an enteric virus, and bacteriophage MS2 (MS2), which was used as a process control. DNA viruses including Salmonella phage p22 and crAssphage, recently classified within the genus *Carjivirus*, were also evaluated. Both spiked samples and concentrated wastewater samples were analyzed.

HTP treatment (120 °C, 15 s) resulted in distinct changes in measured viral genome concentrations depending on the virus species (Table 5). Among RNA viruses, PMMoV in environmental wastewater samples showed the largest increase of approximately 3.5 log_10_ (~ 3000-fold) following HTP treatment. CGMMV detected in environmental samples showed an increase of ~ 2.4 log_10_ (~ 270-fold) following HTP treatment. Similarly, the spiked RNA virus MS2 showed an increase of ~ 1.6 log_10_ (~ 38-fold).

**Table 5:** Viral concentrations in concentrated wastewater samples under HTP (−) and HTP (+) conditions by genome type and sample type.

Virus	Genome type	Sample type	HTP (−) (log_10_ gene copies/μL)	HTP (+) (log_10_ gene copies/μL)
PMMoV	RNA	Environmental	1.0 ± 0.1	4.5 ± 0.1
GII	RNA	Environmental	ND	0.4 ± 0.7
CGMMV	RNA	Environmental	1.3 ± 0.1	3.7 ± 0.2
MS2	RNA	Spike	8.4 ± 0.2	10.0 ± 0.2
p22	DNA	Environmental	1.7 ± 0.3	2.0 ± 0.1
crAssphage	DNA	Environmental	2.7 ± 0.2	2.7 ± 0.2

Values are presented as mean ± SEM (n = 5). ND: not detected. Environmental: viruses present in environmental wastewater samples. Spike: viruses spiked into samples prior to analysis. MS2 concentrations were estimated based on plaque assay-derived values by preparing serial dilutions of the input stock.

For the enteric RNA virus GII, viral genomes were not detected under HTP(−) conditions but were detected under HTP(+) conditions, with measured concentrations in the low-copy range. In contrast, DNA viruses showed limited changes following HTP treatment. The environmental DNA virus p22 exhibited an increase of ~ 0.3 log_10_ (~ 1.8-fold), while crAssphage showed minimal difference between HTP(−) and HTP(+) conditions (~ 0.03 log_10_, ~ 1.1-fold).

These results indicate that the magnitude of change in measured viral genome concentration following HTP treatment differed across virus species under the tested conditions.

## Discussion

HTP is a physical lysis method originally developed to disrupt cells, bacteria, and fungi through rapid heating and pressurization. Heat-based physical lysis methods have been widely applied to disrupt bacterial and mammalian cells, as reviewed by Islam et al.^15^ and Zhao et al.^16^. These approaches support the rationale for applying high-temperature pressure lysis to viral particles. This study evaluated the applicability of HTP for direct quantification of viral genomes in environmental samples, with the aim of simplifying the virus detection workflow by eliminating the RNA extraction step.

Using PMMoV as a model RNA virus, HTP at 120 °C for 15 s yielded quantification results comparable to those obtained by conventional RNA extraction. Both one-step and two-step RT-qPCR assays demonstrated less than one order of magnitude difference compared to AllPrep-purified RNA, showing that the overall quantification performance was comparable. In addition, dPCR confirmed the consistency and reliability of quantification from HTP-processed samples. Furthermore, serial dilution experiments using purified PMMoV confirmed that the HTP method enables consistent detection down to 1 copy/μL by both RT-qPCR and dPCR. Although increased variability was observed at lower concentrations, detection was reproducible across all tested levels, supporting the practical sensitivity of the method. These findings demonstrate that, despite increased variability at low concentrations, the HTP method provides reliable and sensitive quantification down to 1 copy/μL, as confirmed by both RT-qPCR and dPCR.

Compared to other extraction-free approaches^7–9^, HTP offers a more physically controlled and reproducible lysis mechanism. Rapid heating increases the atmospheric pressure inside the tube, which is presumed to facilitate viral particle disruption.

The composition of wastewater matrices can contain surfactants and other inhibitory substances that potentially affect lysis efficiency and downstream qPCR performance^5,6^. However, the results of this study suggest that the difficulty in quantifying PMMoV from non-concentrated wastewater may be associated, at least in part, with low viral abundance. When purified PMMoV was spiked into tertiary-treated wastewater, matrix effects appeared to be limited, and no significant inhibition of qPCR amplification efficiency was observed following HTP treatment. Although some complex components of tertiary-treated wastewater may remain after processing, they did not substantially interfere with downstream reactions under the tested conditions.

RNA integrity analysis using a Bioanalyzer indicated that viral genomes may be degraded following HTP treatment, particularly at temperatures above 140 °C. This observation aligns with the finding that the optimal condition for HTP is 120 °C and supports the validity of this temperature setting.

HTP-induced RNA fragmentation may introduce bias in quantification depending on the target region within the viral genome. Therefore, primer and probe design should prioritize short amplicons and regions less susceptible to fragmentation. However, the consistency of fragmentation patterns across different viruses and experimental conditions remains unclear and warrants further investigation. Since the extent of RNA fragmentation and its impact on quantification may vary depending on viral genome structure and primer design^10^, further validation using a broader range of primer sets and environmental viruses is warranted.

Because the synthetic oligonucleotides used in this study targeted only a specific genomic region, using oligonucleotides or primer sets that cover a broader portion of the PMMoV genome could enable a more comprehensive assessment of HTP-induced RNA fragmentation.

Noroviruses and enteroviruses more directly reflect acute infection dynamics; however, their concentrations in wastewater exhibit large fluctuations and often fall below the detection limit during non-epidemic periods^17^. On the other hand, PMMoV is consistently present at high and stable concentrations in wastewater, making it widely used as a reliable fecal indicator^1,2^. Moreover, recent studies have shown that it can serve as a normalization marker for interpreting viral signals in wastewater^18^. However, PMMoV may not fully represent the behavior of more fragile or less abundant enteric viruses. In this study, the enteric RNA virus GII was also evaluated; however, it was present at low levels in the tested environmental samples, and thus only limited observations could be obtained. Nevertheless, the attempt to include an enteric virus provides preliminary insight into the potential applicability of HTP treatment beyond highly abundant targets. Our findings suggest that the effect of HTP treatment may vary across different virus species. This is supported by the data obtained from additional analyses including RNA viruses (CGMMV, MS2, and GII) and DNA viruses (p22 and crAssphage). RNA viruses generally showed larger increases in measured concentrations following HTP treatment (e.g., ~ 2.4 log_10_ for CGMMV and ~ 1.6 log_10_ for MS2), whereas DNA viruses exhibited relatively limited changes between HTP (−) and HTP(+) conditions under the tested conditions. The reasons for these differences remain unclear. However, multiple factors, including differences in genome type, viral structure, and genome accessibility, may influence the response to HTP treatment.

Extending HTP to other pathogenic microorganisms—including bacteria and fungi—may further demonstrate its value as a broadly applicable lysis approach, supported by evidence that short, high temperature treatments enable direct nucleic acid detection across diverse microbes without conventional extraction^15,19^. In addition to viruses, HTP-based approaches could also facilitate rapid microbial analyses in environmental and clinical contexts, as suggested by extraction free or thermal lysis workflows for bacteria and heat shock/direct PCR protocols demonstrated for filamentous fungi^20,21^.

It should be noted that all viruses evaluated in this study were non-enveloped. Therefore, the applicability of HTP to enveloped viruses remains to be determined. Enveloped viruses may respond differently to thermal and pressure stress due to the presence of a lipid membrane, which could influence lysis efficiency and nucleic acid stability. However, further investigation including enveloped viruses is required to clarify the scope of applicability of this method.

In addition, the number of replicates used to determine the limit of detection (LOD) was relatively small (n ≤ 6), which may affect the robustness of the estimated sensitivity. Therefore, the reported LOD should be interpreted with caution, and further validation using larger sample sizes would be beneficial.

Several recent studies have proposed portable or field-deployable virus detection systems, such as Bento Lab and PicoGene PCR1100 for surface sampling^22^, the QPsor system combining Nanotrap particles and isothermal amplification^23^, and CRISPR-based WATER NEWS for wastewater surveillance^24^. While these systems offer rapid detection, they often require specialized reagents, complex workflows, or proprietary devices. In contrast, the HTP method demonstrated in this study provides a simplified workflow without nucleic acid extraction, while remaining compatible with standard qPCR workflows, potentially supporting scalable environmental virus monitoring applications.

In conclusion, the HTP method, when combined with qPCR, was demonstrated to be applicable for rapid virus quantification. Although the device is not commercially available at present, the treatment conditions, including temperature and duration, are reproducible, and efforts toward standardization are currently underway. Future research is expected to focus on expanding the applicability of HTP to a wider range of virus types and environmental matrices, as well as integrating the method into automated platforms for real-time and on-site virus surveillance.

## Methods

### Virus

PMMoV, obtained from the Katayama Laboratory (University of Tokyo), were resuspended in 25 mM Tris–HCl (pH 8.0) to prepare a viral standard solution.

### Virus concentration

Tertiary-treated wastewater samples from the Aqua Nueva Water Reclamation Facility were collected at the University of Arizona and concentrated from 100 mL to approximately 200 μL using the FluidPrep™ Concentrating Pipette System and CPT-Ultra-Irradiated tips (InnovaPrep, USA). The procedures were conducted according to the manufacturer’s manual, and the protocol followed was the 'Protocol COVID WW’ provided by InnovaPrep^25^.

### RNA extraction and viral lysis

For conventional genome extraction, viral RNA was purified using the AllPrep kit (QIAGEN, Germany). The high-temperature and high-pressure treatment device (HTP device) used in this study was developed in-house by Yokogawa Electric Corporation (Japan) (supplemental Fig. S1) and consists of a dry block heater (PA3003; MSA Factory, Japan). Samples were placed in high-temperature and pressure-resistant tubes made of Veradel® (polyethersulfone; 250 μL capacity; Solvay S.A., Belgium), and sealed with caps equipped with O-rings. These sealed tubes were heated at temperatures ranging from 80 to 180 °C for durations between 5 and 60 s to prepare the viral lysis solution. The internal pressure generated upon heating was preserved by sealing the tubes.

### qPCR

The primers and probes used for quantitative PCR targeting PMMoV, NV GII (GII), CGMMV, p22, and crAssphage are listed below.

PMMoV: Forward primer PMMoV-F (5′-GAGTGGTTTGACCTTAACGTTTGA-3′), Reverse primer PMMoV-R (5′-TTGTCGGTTGCAATGCAAGT-3′), Probe PMMoV-P (5′-FAM-CCTACCGAAGCAAATG-MGBNFQ-3′).

NV GII (GII): Forward primer GII-F (5′-CARGARBCNATGTTYAGRTGGATGAG-3′), Reverse primer GII-R (5′-TCGACGCCATCTTCATTCACA-3′), Probe GII-P (5′-FAM-TGGGAGGGCGATCGCAATCT-TAMRA-3′).

CGMMV: Forward primer CGMMV-F (5′-GCATAGTGCTTTCCCGTTCAC-3′), Reverse primer CGMMV-R (5′-TGCAGAATTACTGCCCATAGAAAC-3′), Probe CGMMV-P (5′-FAM-CGGTTTGCTCATTGGTTTGCGGA-TAMRA-3′).

MS2: Forward primer MS2-F (5′-GTCCTGCTCRACTTCCTGT-3′), Reverse primer MS2-R (5′-CGGCTACCTACAGCGATAG-3′), Probe MS2-P (5′-FAM-CAWGGTACGATCTCGCTAAAGACATTA-MGBN-3′).

P22: Forward primer P22-F (5′-CTTAACAAGCTCTGACTGCTCATCA-3′), Reverse primer P22-R (5′-CCATCGCCTGTGACTGGAT-3′), Probe P22-P (5′-FAM-TCGCAACGATGCAGAACGACTCG-TAMRA-3′).

crAssphage: Forward primer crAss-F (5′-CAGAAGTACAAACTCCTAAAAAACGTAGAG-3′), Reverse primer crAss-R (5′-GATGACCAATAAACAAGCCATTAGC-3′), Probe crAss-P (5′-FAM-AATAACGATTTACGTGATGTAAC-MGB-3′).

One-step RT-qPCR was performed using One Step PrimeScript™ III RT-qPCR Mix (Takara Bio, Japan). The thermal cycling conditions were as follows: reverse transcription at 52 °C for 5 min, initial denaturation at 95 °C for 2 min, followed by 40 cycles of 95 °C for 10 s and 60 °C for 30 s. Two-step RT-qPCR was conducted using the High-Capacity cDNA Reverse Transcription Kit with RNase Inhibitor (ThermoFisher, USA) for reverse transcription, followed by qPCR using TaqPath™ qPCR Master Mix CG (ThermoFisher, USA).

### qPCR standard preparation and qPCR instrument

For qPCR standard preparation, a synthetic oligonucleotide representing a 208-base pair region of the PMMoV genome (nucleotides 1808–2015) was synthesized by Eurofins (Japan). The DNA fragment was serially diluted to known concentrations for use as a quantification standard. For other viral targets (GII, CGMMV, and p22), standard materials were prepared using either synthetic DNA fragments or viral RNA standards corresponding to the target regions, and serially diluted for quantification. For MS2, viral concentrations were determined based on plaque assay results, and the corresponding values were used for quantification. Amplification efficiency was calculated from the slope of the standard curve. qPCR assays were performed using the Thermal Cycler Dice (Takara Bio, Japan).

### Digital PCR (dPCR)

Digital PCR was performed using the QIAcuity Digital PCR System (QIAGEN) with a two-step protocol. Reactions were prepared according to the manufacturer’s instructions using the QIAcuity 4× Probe PCR Master Mix and loaded into 24-well QIAcuity Nanoplates. cDNA synthesis was conducted as described in the qPCR section. The same primer and probe sequences used in the qPCR assay were applied, with the probe labeled with FAM.

Each run included no-template controls (NTC). Thermal cycling was carried out on the QIAcuity instrument under the following conditions: initial denaturation at 95 °C for 2 min, followed by 40 cycles of denaturation at 95 °C for 15 s and annealing/extension at 60 °C for 60 s. Fluorescence signals were detected using the QIAcuity Software Suite. Thresholds were determined based on control wells, and absolute quantification was performed automatically by the software based on the Poisson distribution.

### Determination of LOD and LOQ

The limit of detection (LOD) was defined as the lowest concentration at which measurable signals were reproducibly detected across replicate measurements and could be distinguished from the negative control. The limit of quantification (LOQ) was defined as the lowest concentration at which quantification showed acceptable repeatability, based on a coefficient of variation (CV) of ≤ 25%. LOD and LOQ were determined using serial dilution experiments of PMMoV (see Supplementary Table S1).

### Assessment of RNA integrity after HTP

To evaluate the integrity of viral RNA following HTP treatment at various temperatures, samples were analyzed using the Bioanalyzer system (Agilent Technologies, USA). RNA preparation and analysis were performed according to the manufacturer’s instructions using the RNA 6000 Pico Kit. Electropherogram profiles were examined to assess the extent of RNA fragmentation under different HTP conditions. All reagents and consumables were stored at 4 °C until use.

### Statistical analysis

Statistical analyses were performed using Student’s *t*-test and one-way ANOVA. Results are presented as mean ± standard error of the mean (SEM).

## Supplementary Information

Below is the link to the electronic supplementary material.

Supplementary Material 1

## Data Availability

All data generated or analyzed during this study are included in this published article and its supplementary information files.
