# Supplementary material for: Direct quantification of waterborne viruses via high-temperature and high-pressure treatment: a simplified nucleic acid extraction-free approach
Source: Sci Rep. 2026 Jun 21;16:20119. doi: 10.1038/s41598-026-57432-2 (PMC13324460; doi:10.1038/s41598-026-57432-2)
Supplement: Supplementary file 1 — Supplementary Material 1 [file 41598_2026_57432_MOESM1_ESM.pdf]

Supplementary Information

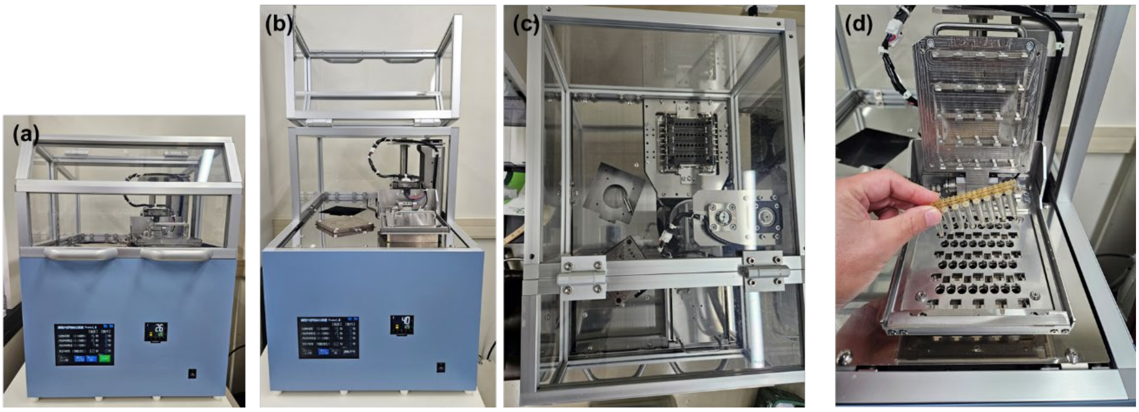

Supplementary Figure S1. Overview of the High Temperature Pressure (HTP) system. (a) Front view with lid closed. (b) Front view with lid open. (c) Top view of the system. (d) Close-up of the tube holder inside the chamber shown in (c). The hand is holding an HTP tube, which is being positioned for insertion into the designated holes in the holder.

Table S1. Serial dilution data for PMMoV quantification used to assess assay sensitivity.

Concentration (copies/µL)	n	RT-qPCR mean	RT-qPCR SEM	dPCR mean	dPCR SEM
H_2O	6	1.6	0.8	0.3	0.1
1.0	6	3.2	1	0.9	0.5
10.0	3	5.4	0.6	10.3	0.9
100.0	3	59.3	8.4	112.8	1.1
1000.0	3	609	20.9	916	18.7
10000.0	3	5104.2	227.3	6301.1	305.1

Values are presented as mean ± SEM (gene copies/µL). Spiked samples were prepared by adding known concentrations of purified PMMoV to buffer. H_2O samples served as negative controls without viral input.
